# Managing mental incapacity in the 20th century: A history of the Court of Protection of England & Wales

**DOI:** 10.1016/j.ijlp.2019.101524

**Published:** 2020

**Authors:** Janet Weston

**Affiliations:** Centre for History in Public Health, London School of Hygiene and Tropical Medicine, 15 -17 Tavistock Place, London WC1H 9SH, UK

**Keywords:** Mental capacity, Court of Protection, Mental illness, Disability, History

## Abstract

This article explores the history of the Court of Protection of England & Wales (CoP) over the twentieth century. The CoP, which is responsible for making financial and welfare decisions on behalf of those deemed incapable of doing so themselves, presently faces a rapidly growing caseload, and considerable scrutiny and critique. Such close attention to its work may be new, but many of the issues it faces have deep roots. Using practitioners' texts, judgements, and the archives of the CoP and the Lord Chancellor's Office, I review the evolution of the CoP in terms of its structure and caseload, its decisions regarding incapacity, its efforts to manage the affairs of those found incapable, and its long-term survival. This reveals the origins of many of the issues it faces today, the different anxieties and approaches that have animated its work in the past, the ways in which approaches to incapacity have changed, and the value of a historical perspective.

## 1 Introduction

The role of the Court of Protection of England and Wales (CoP), according to its website, is to ‘make decisions on financial or welfare matters for people who can't make decisions at the time they need to be made (they “lack mental capacity”).’ This weighty responsibility and wide-ranging power in relation to some of society's most vulnerable has attracted considerable attention over the last decade. According to an assortment of newspaper articles, this is ‘the most sinister Court in Britain’, ‘Britain's most secret court’, and the ‘secret court of living hell’, with ‘the power of life or death’ (Beckford, 2010; Booker, 2015, Booker, 2016; Lewis, 2009). It has attracted less shrill but much more searching scrutiny and critique from academic circles, with a profusion of research into the court's activities and associated issues of determining mental incapacity, autonomy, best interests, participation, supported decision-making, transparency, and the United Nations Convention on the Rights of Persons with Disabilities (Clough, 2018; Coggon, 2016; Craigie & Davies, 2019; Kong, 2017; Ruck Keene, Kane, Kim, & Owen, 2019; Series, 2016; Series & Nilsson, 2018).^1^ The CoP has also been criticised by the UK Parliament (House of Lords Select Committee on the Mental Capacity Act 2005, 2014) and in trade publications (Sofaer, 2010), primarily for being expensive, slow, and inefficient, while the financial abuse of those who lack capacity remains a concern (Dalley & Gilhooly, 2017; Terrell, Bielanska, Holmes, & Frimston, 2018, p. viii). All the while, Ministry of Justice statistics for the Family Courts show that the CoP's caseload is increasing. This area of the law clearly presents problems both practical and conceptual in nature, which are affecting growing numbers of people.

The present Court of Protection was established in 2007 under the Mental Capacity Act, 2005 (MCA). An attempt to tell its twentieth century history may therefore seem strange, but in fact the CoP pre-dates the MCA in name, concept, and in substantive parts of its role. This is sometimes acknowledged in textbooks and wider discussion (Terrell et al., 2018, p. 46), but is more often obscured by perceptions of the MCA as a watershed moment. The MCA, as former Senior Judge and Master of the CoP Denzil Lush has remarked, was wrongly viewed by some as a ‘blank canvas’ onto which a brand new approach to mental capacity could be painted (Lush, 2018). This perspective, in which the history of the CoP is overlooked, reflects the fact that the CoP was not at any time during the twentieth century particularly well-known or high-profile. Its decisions were rarely reported, it was not the target of the century's mental health legislation, and its activities provoked virtually no controversy or public attention. Yet, this little-known institution became the cornerstone of contemporary mental capacity law and practice. Its survival, in essence if not in exact replica, makes England & Wales the only jurisdiction with a specialist court of this kind for mental capacity decisions (Ruck Keene et al., 2019, p. 59). What did the ‘old’ CoP do, and why did it endure? How did it tackle the problems of its own time, many of which resonate strongly with those of today? By offering a history of this institution over the twentieth century, I hope to show both the deep roots of some of the issues currently facing the CoP, and the rather different anxieties and approaches to incapacity that have animated its work in the past.

Beyond these specific concerns, a short history of the CoP fulfils two further goals. Firstly, it offers insights into the evolution of mental health law in England & Wales, and the idea of mental capacity itself. Histories of psychiatry often focus on hospitals, admissions, and the statutes that govern them, but I show that attention to other aspects of historical mental health law such as incapacity reveal patterns of stasis and change that would otherwise go unnoticed. Legal scholarship on mental capacity sometimes looks to history to point out patterns and diversions in how the needs of persons with disabilities or doubtful capacity have been tackled across time (Allen, 2015). The past has also been mined for specific solutions, when legal difficulties seem to reach crunch-point (Hoggett, 1988; Ward, 1990). But, as Peter Bartlett has observed, our understanding of mental capacity decisions themselves has been limited by the fact that there was ‘no general mechanism to report those found to be lacking capacity, nor in general to monitor the decisions being made about these people’ (Bartlett & Sandland, 2000, p. 349). Such decisions were diffuse and thus difficult to locate, with the result that the meaning and uses of the concept have remained elusive. But by focusing on the history of the CoP, where determinations of mental capacity were consistently being made, we can begin to develop a richer understanding of the concept, how it was handled, and what has changed or stayed the same over time. This history is accessible through practitioners' textbooks, the few available reported and unreported judgements, administrative records, and especially through the archive of case files and administrative records available at the National Archives in London.^2^ This attention to the history of incapacity shows that today's approaches are not always as novel as their proponents sometimes imply, and – in case there remained any doubt – confirms that ‘mental incapacity’ is not and never has been an objective truth to be uncovered.

Secondly, this article engages with current debate about the role and value of history. As Russell Sandberg has recently argued, legal history has considerable radical potential. It can demonstrate that ‘legal institutions are not fixed, that every line drawn in the law and everything the law holds as sacred is arbitrary’ (Sandberg, 2018, p. 24). This article takes up this challenge, using a variety of sources including those not typical to legal history, introducing new voices and perspectives, and assessing the causes of change and its absence. It also demonstrates that an understanding of the law demands knowledge of its operation in context, linking the doctrinal with the socio-legal (Lacey, 2001). In relation to mental incapacity, we see that changes in legal practice are often accidental and unplanned, and that legal approaches are driven by medical, social, economic, and practical contexts. I also seek to demonstrate that this kind of critical legal history can be applied to the recent past as well as to more distant periods. Firstly, though, it is useful to look slightly further back in time to provide a short explanation of how the ‘old’ Court of Protection came to be.

## 2 The background to the Court of Protection

The origins of the CoP can be traced to a royal prerogative, the evolution of which has been well summarised elsewhere (Bartlett & Sandland, 2014; Lush, 2014; Stebbings, 2012; Theobald, 1924; Unsworth, 1993). The prerogative confirmed that the monarch exercised authority over the property of ‘lunatics’ and ‘idiots’. Over time, this authority was delegated to the Lord Chancellor, and from him to the Judge in Lunacy, and by the late nineteenth century, to the two Masters in Lunacy (who became one in number in 1923). By the 1800s, it was enacted through a Commission in Lunacy which could be instigated by any interested party, but usually a family member of the alleged lunatic. This would prompt an event called an inquisition. The outcome of the inquisition could be determined by the Judge or Master alone, or heard before a jury with witnesses called and questioned. This latter option became increasingly rare. If the person were found by inquisition to be lunatic or idiot, a ‘committee of the estate’ – again, often a family member – was appointed to look after their property. There might also be a ‘committee of the person’, appointed to be responsible for the individual's personal welfare. This separation within the incapacity jurisdiction between property and personal welfare became significant in the late twentieth century.

Over the nineteenth century, procedures surrounding Commissions in Lunacy were adjusted and refined. Important changes included the creation of the Lord Chancellor's Visitors in 1833, positions to be held by two senior doctors and a barrister of at least 10 years standing. The Visitors were tasked with monitoring those under the guardianship of a committee, and providing reports to the Office of the Masters in Lunacy. Further adjustments included changes to the powers of the Judge and Masters in Lunacy to enable more streamlined estate management and abbreviated forms of inquisition for less affluent individuals, although Commissions in Lunacy remained expensive and impractical for all but the fairly well-to-do. Cost was not the only disincentive to this type of intervention. Much like their successors today, Masters in Lunacy laboured under the disadvantage of being perceived as secretive and mysterious. At the same time, since inquisitions before a jury were open to all, they could involve the extremely public airing of personal and family matters. Occasionally, inquisitions with juries such as the case of William Windham in 1861 attracted considerable publicity and scrutiny (Degerman, 2019; Heaton, 2012; Jones, 1971; Taylor, 1883). This unwelcome attention, in combination with the boom in asylum populations over the nineteenth century, meant that the number of inquisitions was declining.

The growth of the asylum population had a twofold impact upon inquisitions. Firstly, it meant that opportunities for the kind of questionable decision-making that might prompt a Commission in Lunacy, such as William Windham's gifts of extremely expensive jewellery, were more easily controlled by alternative means. After all, individuals who were lawfully detained as persons of unsound mind could find their access to both money and people greatly restricted in a day to day sense, meaning that more formal steps to regulate their financial habits were often unnecessary. Secondly, it prompted the beginnings of an alternative structure within which decisions about mental capacity and financial management could be made. This began with the Lunacy Regulation Act of 1862, which provided that the Lord Chancellor could ‘make [an asylum patient's] property available for the patient's benefit or maintenance. It could be sold and the proceeds paid to a relative or other proper person to apply under the court's direction’ (Stebbings, 2012, p. 396). The party receiving funds to manage in this way was known as a receiver. The receiver had no formal control over the person, only their property, and was answerable to the court. Importantly, this process did not require an inquisition, as the individual in question had already been detained as a person of unsound mind. Their inability to manage their affairs was assumed.

Additional alternatives to inquisitions were introduced by the 1890 Lunacy Act. Historian of psychiatry Akihito Suzuki has argued that this Act created ways for people to avoid Commissions of Lunacy (Suzuki, 2006). One such way for those detained in hospital had been provided in 1862, as outlined above, but an inadvertently revolutionary aspect of the 1890 Lunacy Act was to make this available for anybody at all, even if they were *not* detained as a person of unsound mind. To do this, section 116 (d) of the 1890 Act empowered the Judge and Masters in Lunacy to appoint a receiver to manage the affairs of anyone, if they were satisfied that the individual was incapable of managing their affairs ‘by reason of infirmity caused by disease or age’. Thus, in order to place an individual's affairs in the care of a receiver, it was no longer necessary to for them to be lawfully detained or to undergo a potentially public (and expensive) inquisition. The primary intention behind section 116 (d) had been to protect the estates of those who suffered from mental infirmity due to old age or ‘excess’ (Heywood and Massey, 1907, p. 90; Theobald, 1924, p. 6), but, as we will see, it was interpreted far more widely in the twentieth century than its drafters had likely imagined.

Although receivers and committees of the estate initially had slightly different powers, these had been equalised by the early twentieth century. The process of applying for a receiver was faster, easier, and cheaper than a Commission in Lunacy. Unsurprisingly, it became the more popular option. In 1890 there were over 1000 lunatics so found by inquisition, but by 1922 this number had dwindled to less than 300 (Theobald, 1924, p. 80). By 1954, the inquisition procedure was ‘nearly obsolete’, recommended only on rare occasions to meet the requirements of foreign jurisdictions, to prevent someone from marrying, or to exercise another form of restraint or control over the person such as to ‘compel a patient to accept care or treatment’ outside a hospital (HMSO, 1957, p. 267; Hunt & Phillips, 1954). The option of an inquisition (and of a committee of the person) was effectively removed once and for all by the Mental Health Act of 1959, although the last person to have been the subject of an inquisition died as recently as March 2018 (*In the matter of A* [2018]). While inquisitions and committees of the person or estate fell into obsolescence, the number of receivers rose steeply. It was therefore mostly the appointment, oversight, and discharge of receivers that occupied the Masters in Lunacy during the twentieth century. And it was their office, known variously as the Office of the Masters in Lunacy, Lunacy Office, and Management & Administration Department, which was to become the Court of Protection.

### 2.1 From lunacy to protection

The term ‘lunacy’ was falling out of official use in the early twentieth century. The Rules in Lunacy dating from 1892 laid out forms that avoided the word, particularly for those coming under the jurisdiction of the Masters in Lunacy under the terms of section 116 (d) of the 1890 Act: those who had *not* been found lunatic at inquisition or detained as persons of unsound mind, but were nevertheless deemed incapable due to ‘disease or age’. These individuals were perhaps not lunatics at all, it was acknowledged, but simply elderly or mentally frail. In any case, it was ‘not thought desirable’ to publicise the condition of those coming under the aegis of the Lunacy Office in quite such stark terms (*Re Browne* [1894], 415). Complaints were reportedly raised that it was injurious to those struggling with mental infirmity to receive documents headed ‘In Lunacy’, and there are examples in the archives of attempts to conceal such papers from the individuals they described because of the distress that the word ‘lunacy’ might cause (TNA J92/22). The word was quietly dropped from ever-more items of paperwork, but its disappearance in the 1920s from the Office letterhead caused problems. The Office of the Master in Lunacy became, simply, the Office of the Master. Located at the Royal Courts of Justice, where there were many other Masters to be found, correspondence so addressed was liable to go astray. Gerald Hildyard KC, who held the position of Master in Lunacy from 1923 to 1928, proposed ‘Management and Administration Department’ as a practical alternative name for his office in 1925. With some satisfaction, he pointed out that the M.A.D. retained a reference to lunacy in its initials (TNA LCO 4/53).

Hildyard seems to have been alone in his approval of this new name. His successor as Master, Henry Methold KC, found the acronym ‘more apt than happy’ and colleagues complained that the full name was cumbersome, confusing, and even downright misleading (TNA LCO 4/53). Methold proposed a name for his department that reflected its ancient lineage, along the lines of the ‘Court of the King's Wards’, but this proposal was not very warmly received. One repeated concern was that children who were wards of court might be tainted with connotations of mental illness (TNA LCO 4/53). Stigma was clearly still present. Chancery Judge Sir Albert Clauson suggested something including the word ‘protection’, such as ‘Court of Protection’, and this was finally adopted in 1947 under the Patients' Estates (Naming of Master's Offices) Order. ‘Court of Protection’ never generated any great enthusiasm, though. It was, simply, the only suggestion to which nobody objected very strongly. As the Master of the Rolls observed when the new name was agreed, ‘I have racked my brains but for the life of me I can think of nothing better…. the perfect word probably doesn't exist’ (TNA LCO 4/53).

These changes of name did not themselves herald new structures or practices, but they were not without meaning. ‘Lunacy’ disappeared as ideas of mental illness changed and a wider variety of mental infirmities were recognised (Long, 2014; Takabayashi, 2017; Westwood, 2010). New medical approaches reflecting this shift took the form of clinics for the early and voluntary treatment of nervous or functional disorder. These began to emerge in the early twentieth century and were then encouraged by the recognition and treatment of shell shock during and after the First World War (Barham, 2004; Loughran, 2017; Weston, 2017 chapter 2). Attention to the impact of stigma surrounding mental illness, as well as the existence of a wide variety of disturbed states of mind that did not fit the mold of ‘lunacy’, meant that the term ‘lunacy’ itself became less common in medical and policy circles, and the word ‘lunatic’ was prohibited from any statutory enactments or subordinate documents by s.20 of the Mental Treatment Act of 1930. It is notable that the field of mental capacity law and practice was an early adopter in this respect, avoiding the term and adopting a broader view of those for whom it should be responsible as early as the 1890s.

That said, the adoption of the prosaic ‘Management and Administration Department’ indicates a lack of concern for status and external recognition. It is perhaps unsurprising that this name was proposed and implemented by the shortest-serving Master in Lunacy. Master Hildyard may have been less interested in the status of the department than some of his longer-serving colleagues and successors, and less aware of the value of asserting the importance of his department. This was, after all, a niche and low status matter, and the Lunacy Office was an unusual and somewhat anachronistic entity which had been under pressure to merge with the Chancery Division for decades (Stebbings, 2012; Theobald, 1924, pp. 77, 83–84). Insistence following Hildyard's departure that the Department should become the ‘Court of the King's Wards’, an altogether grander option, suggests a sensitivity to status and survival. So, too, did the dogged determination to include the word ‘Court’ in place of ‘department’ or ‘office’. This was a strategic move with long-term consequences. All could agree in 1947 that for ‘certain purposes the Master in Lunacy is a court and has been so held’ (TNA LCO 4/53), even though it was an office in which the vast majority of decisions were made, not by judges, but by the Master and Assistant Masters, not all of whom were qualified legal practitioners. The notion of a ‘court’ run by civil servants became troublesome as its work attained a much higher public profile at the end of the century (Law Commission, 1995, 2.50). Nevertheless, the idea that these matters should indeed be handled by a separate and specialist entity was sufficiently well established for the CoP to be reconstituted in 2007 as a superior court of record.

Including the word ‘protection’, as well as ‘court’, was also strategically wise. It brought suggestions of safeguarding and support, which tallied with the expanding welfare state of the 1940s. Indeed, Assistant Master Ronald Poyser, who pressed most energetically for the change of name in 1947, firmly believed that the Court of Protection should be viewed as a branch of this new welfare state (TNA LCO 2/7695). ‘Protection’ was also associated in the minds of 1940s judges and civil servants with finance in the form of protective tariffs (TNA LCO 4/53). ‘Court of Protection’ thus drew discreet attention to the property itself that was to be protected, rather than the receivers to be supervised or administration undertaken. After all, it was the scale of this property and the complexities involved in its management that had helped to ensure that survival of the Office in the nineteenth century (Stebbings, 2012). It oversaw property worth over £4 million in 1905 (HMSO, 1908, p. 263), equivalent to something like £314 million today.^3^ A new name hinting at such significant responsibilities may have helped to cast the Court of Protection as a distinctive and valuable institution, also playing some small part in its remarkable survival.

## 3 The Court of Protection caseload

The Court of Protection acquired a few new names over the first half of the twentieth century, then, but hereafter I will refer to it as the CoP for simplicity and brevity. On the surface, little else was changing. The 1959 Mental Health Act was later identified as significant for mental incapacity law (Law Commission, 1995, 2.20), by placing the CoP on an entirely statutory footing and revoking the royal prerogative, which removed its formal authority over personal welfare matters. Following further restrictions to guardianship powers in the 1980s, this created a legal lacuna regarding decisions about medical treatment on behalf of those lacking capacity. But this was an unintended outcome: the 1959 Act was not designed with any material effects on the CoP in mind (TNA LCO 2/7700). Indeed, the CoP was not the primary target of this Act or the other mental health legislation of the century. It encountered no major scandals, restructures, or other signs of upheaval. There were very few reported judgements from the CoP itself or appealed therefrom. This peaceful façade has perhaps given the impression of stasis and helped to render the ‘old’ CoP invisible. Yet, its work, its decisions, and the people with whom it dealt were all undergoing change.

**Fig. 1:**
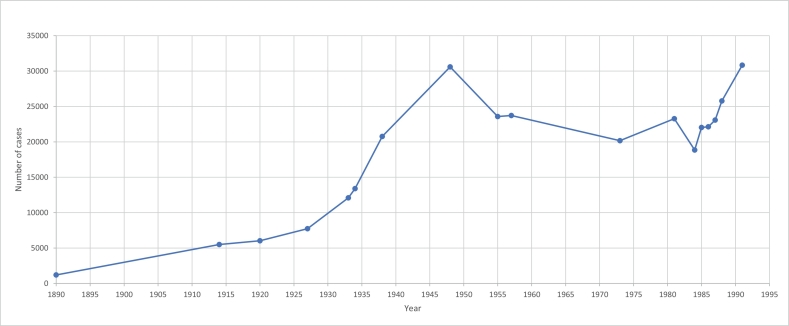
Open cases with the Court of Protection.

The first change to record is the number of individuals under its jurisdiction [Fig. 1]. A gradual increase in applications to the CoP over the early 1900s reflected an equally gradual spread of awareness amongst solicitors, bank managers, doctors, local welfare officers, and others, that there was an alternative to the costly inquisition following the Lunacy Act of 1890, and that the affairs of a person of doubtful capacity should not be ignored or left in the hands of unofficial helpers (Mills & Poyser, 1934). Applications alone approached 1000 during 1922 (Theobald, 1924, p. 103). The number of individuals with receivers overseeing their affairs then underwent an extremely rapid rise over the 1920s and 1930s, doubling in the 12 years to 1934, and more than doubling again in just four more years (TNA LCO 4/49, TNA LCO 4/55). There was a distinct decline over the 1950s (HMSO, 1957, p. 291), which went into reverse at some point around the late 1960s or early 1970s (House of Commons, 1974). Receiverships had therefore peaked in the late 1940s in excess of 30,000, a figure that would be reached again towards the end of the century (HC (Written answers) 2 May 1991 vol 190).

The reason for the sharp rise from the 1920s was the cause of some contemporary speculation. In 1954, the seventh edition of the leading practitioners' textbook, *Heywood & Massey's Court of Protection Practice,* suggested that this ‘great increase’ had been down to ‘social changes resulting in redistribution of wealth’ (Hunt & Phillips, 1954, p. 3). In other words, more people were coming to the CoP because more of the population had assets that might need protecting. This passing observation raises intriguing questions about contemporary perceptions of welfare and taxes, and indeed, whether the authors were correct to understand that wealth had been redistributed to a markedly different degree in the preceding decades. Setting these questions aside, research elsewhere into income and wealth over the twentieth century has suggested that the numbers of those in the UK who had *no* money or property at all was decreasing over the early twentieth century. Over the first half of the century, for example, a rising percentage of the working classes held money in a savings account of some kind (Atkinson, 2000). More widespread ownership of any property, and particularly property that involved financial institutions with formalised processes for accessing assets, was likely to increase the workload of the CoP.

This was not the only explanation. One CoP clerk speculated in 1934 that the ‘stress of economic conditions during recent years’ might be a contributory factor (TNA LCO 4/48). Master Methold agreed that financial stress was not infrequently a cause of mental breakdown (TNA LCO 4/50). The archive of CoP case files adds further insight along these lines: financial strain within a family often encouraged applications to the CoP to release an infirm person's assets. Mrs. Emily Mathews, for example, contacted the CoP in 1928, two years after her husband Charles had been detained as a person of unsound mind, because she could no longer afford the premiums on their joint life policy with the Prudential. Mrs. Mathews asked to be appointed as her husband's receiver so that the policy could be surrendered, easing the burden on her small income and releasing capital to both her and her husband (TNA J92/103). The idea of a relationship between prevailing economic conditions and applications to the CoP is strengthened by the fact that the number of receiverships rose once again in the 1970s, just as unemployment rates and severe inflation began to take their toll.

This increase over the 1970s was preceded by a period of decline, beginning around the end of the 1940s. This was intimately connected to the arrival of a more comprehensive welfare state and particularly the National Health Service (NHS), although evidence from 1949 suggests that such an impact was by no means anticipated: a 1949 report noted the persistent increase in CoP caseload to date with anxiety, and made no mention of the possible effects of new social care legislation (TNA LCO 4/55). However, the interwar period had seen many applications to the CoP initiated or encouraged by local welfare officers, guardians of the poor, and county councils, who had an interest in securing the property of anyone receiving care who might have sufficient means to pay for it. Even when such assets were minimal and required for their owner's immediate personal use, local authorities could and did request that the CoP grant a charge over the estate, so that reimbursement at a future date remained possible (TNA J92/1, J92/157, *re TRM* [1939]). With the arrival of state-funded medical and hospital treatment under the 1946 NHS Act, the question of recouping fees for healthcare was removed. Furthermore, the 1948 National Assistance Act revised the terms under which councils provided residential accommodation for those in need of care, meaning that fees were means tested and charging orders were not, as a rule, granted to local authorities (Hunt & Phillips, 1954, pp. 137–139). One major incentive for intervening in the property of those in hospitals or nursing homes was thus removed. There are also signs that, over the 1950s, as the CoP enjoyed some respite from its previously unrelenting workload, senior staff were anxious not to spread *too much* awareness about its role (TNA LCO 2/5701).

This was only temporary. Steadily rising numbers from the 1970s were interrupted only briefly in the early 1980s, when a momentary drop in applications can be attributed to an increase in fees charged by the CoP (LCO 68/22) and a large amount of housekeeping in 1983 which led to the closure of many dormant files. After this, the number of estates overseen by the CoP increased steadily and steeply. Contemporary explanations in the later twentieth century almost invariably referred to an aging population, more likely to experience senile dementia and then living longer with the condition (LCO 68/22). In light of broader patterns across the century, we might also wonder whether the costs of care, both medical and social, had some impact after the welfare provisions in place over the 1950s were hollowed out. The role of the CoP was expanded in 2007, making direct comparisons of workload before and after that time less meaningful, but the rising number of applications seems to be a continual trend. The impact of future changes in the economy and social care provisions within England & Wales remains to be seen.

## 4 Determining incapacity

As with references in the 1980s to the prevalence of senile dementia, discussions about the CoP's caseload often touched upon the wider context of mental illness and its incidence or identification. In more recent decades, such discussions tend to assume an absolute increase in the number of people unable to manage their affairs due to illness and age, but earlier in the twentieth century commentators were sensitive to the idea that perceptions of mental illness and incapacity, and not prevalence, might be changing. In marked contrast with other contexts, in which tests for capacity were well established, there was no judicial determination of the meaning of ‘incapable of managing their property and affairs’ until 2002 (*Masterman-Lister v Brutton & Co* [2002]). This absence of a formal test for capacity to manage property and affairs left applicants to the CoP, medical advisers, and CoP officials all somewhat freer to apply their own approaches and understandings, interpreting the jurisdiction more widely at some times than others.

In the 1930s, CoP officials considered whether receiverships were increasing thanks to a wider interpretation of mental disorder and defect. This was attributed to the Mental Deficiency Acts of 1913 and 1927 and the Mental Treatment Act of 1930, all of which had expanded the parameters of mental abnormality (TNA LCO 4/50). The effects of a wide interpretation of section 116 (d) of the 1890 Lunacy Act were also acknowledged, since it had come to be used as something of a catch-all provision to enable CoP intervention (Mills & Poyser, 1934, p. 16). Exactly *whose* interpretations were driving this change went unspecified, and the lack of any information about unsuccessful applications makes this hard to pin down. Shifting attitudes surrounding mental illness might have encouraged families to perceive incapacity more readily amongst their members, and might also have made it easier to find supportive doctors prepared to deliver the necessary affidavits of incapacity. Equally, the CoP itself may have looked more favourably upon applications that it would have rejected out of hand in an earlier era. It seems likely that each of these played some role, with the rising CoP caseload over the first half of the twentieth century reflecting a broader idea of what it meant to be incapable to manage one's affairs on the part of the public, the medical profession, and the CoP alike.

In practice, the wide and general terms of the 1890 Lunacy Act were used in the early twentieth century to appoint receivers in cases where partial or actual capacity was acknowledged, hospitalisation was unnecessary, and the individual was able to live otherwise independently. After Miss Francis Raggett was discharged from hospital as ‘recovered’, for example, her doctors felt that she was ‘quite rational’ and able to resume control over her property. However, the Lord Chancellor's Visitor found that she was ‘contented that [her accountant] Mr Day should continue to manage her affairs’, and Miss Raggett lived the remaining nine years of her life in boarding houses and with friends, ‘quite sane’, ‘happy’, and ‘busily employed’, but benefitting from the ‘supervision and help’ of her receiver (TNA J92/54). Mr. Arthur Short also lived independently despite the fact that his mother had been appointed as his receiver on his 21st birthday. He was employed as a mechanic's apprentice and then as a steward on cruise ships. Unlike Miss Raggett, Mr. Short became increasingly dissatisfied with the receivership, and it was acknowledged within the CoP that there was ‘nothing to differentiate him from many other young men whose affairs have not been taken out of their hands’. After eighteen years, he successfully applied to be restored to his property (TNA J92/38).

Further examples include individuals described as addicted to alcohol, weak in character, eccentric, or lacking self-confidence. For the CoP to intervene, there was usually either a large amount of money at stake, or strong suggestions that the individual was already taking decisions that were seen as unwise. This might include evidence of exploitation. Tellingly, most of these cases involved unmarried women, and I have written elsewhere about the role of gender in CoP decisions (Weston, 2019). Miss Alice Stevens had a tendency to run up debts (TNA J127/123); Miss Jean Carr was young, rich, and thought to be at risk from money-grabbing friends and suitors (TNA J92/77). Miss Beatrice Alexander had apparently already fallen victim to a manipulative family who had seized control of her home and money (J92/24–27). As in the case of Miss Raggett, the wishes of the individual were also taken into account, although their authenticity would be scrutinised in cases of suspected duress. Medical evidence was somewhat secondary: it was often necessary for the applicant or the CoP to obtain several medical statements or to negotiate with medical visitors and family to implement and maintain receiverships in these borderline cases. Files contain comments such as ‘fairly normal mentally,’ ‘the only thing I find with her is, she can be dominated by a stronger will’; and even ‘able to manage herself and her affairs’ (TNA J92/38, J127/24, J92/77). Clearly, the CoP was sometimes willing to intervene even when it had reason to believe that illness or incapacity was very slight.

This approach foreshadows recent developments in the use of the High Court's inherent jurisdiction to intervene in the affairs of individuals who are acknowledged to have capacity, but who are seen as vulnerable in the context of specific relationships and circumstances (Szerletics, 2011). It also reflected the survival of an earlier practice, in which intervention was based less upon the identification of a specific degree of incapacity, and more upon the perceived needs of the individual. As the Chief Clerk in the Office of the Masters in Lunacy wrote in 1892, there may be cases where the Judge in Lunacy did not think that proceedings were appropriate ‘though the mental infirmity might be such as fully to support them’. Equally, he went on,

there are cases in which, from even a less degree of mental incapacity, the interests of the alleged lunatic render it imperatively necessary that he, and his property, should receive the protection which it is the peculiar province of the Royal Prerogative to afford.

The vital issue, he clarified, was ‘the benefit and advantage of the particular individual’, (Elmer, 1892, p. 4) and not their mental state. This impulse to intervene on the basis of perceived vulnerability or need, and not on the basis of illness, is a recurring one, albeit one that has sometimes been masked by a statutory emphasis upon impairment (Hall, 2012).

These directions from 1892 also contain some elements of the current functional approach to incapacity, in which an individual's capacity to make decisions must be considered in relation to the complexity and importance of the specific decisions that face them (Ruck Keene et al., 2019, p. 57). This Chief Clerk clearly endorsed attention to a full picture of the individual's situation, and not simply their state of mind. The above examples from the 1920s and 1930s of individuals with both a receiver and some degree of mental capacity indicate that personal circumstances were still weighed in the balance throughout the first half of the twentieth century, just as much as (if not more than) the nature of any disease or ability to make decisions. In these cases, the individual's wishes and personality, the perceived likelihood and impact of bad decision-making, and the risks of financial abuse were all considered.

That said, the extent to which a functional approach was adopted should not be overstated. The complexity of an individual's affairs certainly had some impact on the CoP's decisions, but the early twentieth century also saw clear confirmation that capacity to manage one's affairs was global, and could not be assessed in relation to the specific decisions that needed to be made. In his judgment in *re Walker* in 1905, Vaughan Williams LJ acknowledged that the ‘mere mental capacity’ of a ‘lunatic’ might suffice for them to deal with some aspects of their property, but ‘it is necessary for the protection of lunatics generally that they should be debarred from the exercise of such powers’ (*Re Walker* [1905] 174). The only exception was in relation to making a will: someone with a receiver in place might still have testamentary capacity and could then make a will, since this did not interfere with the receiver's management of their property during their lifetime. Otherwise, if a receiver had been appointed, then the receiver alone could deal with the individual's property, no matter how lucid or capable of making the decision the individual might be. This was upheld in later years (*Re Marshall* [1920]), probably encouraged by the growth of the CoP and the concomitant need for legal certainty surrounding the status of its interventions. It complicates existing narratives of mental health law, which describe a move from state intervention on the basis of classification to a more universalist approach to mental illness (Unsworth, 1987). Seeing incapacity as global was universal inasmuch as all those found incapable were treated as equally and entirely incapable, but it still relied heavily upon the initial classification of incapacity as a result of disease. The impact of this classification or ‘status’ upon an individual's rights became a key criticism of the CoP in the 1980s and 1990s (Law Commission, 1995, pp. 32–33): by the end of the century, such weighty classifications were still alive and well.

There are signs, though, that views of incapacity narrowed somewhat over the second half of the century to exclude those with more minor forms of mental illness or learning disability. This is difficult to trace, as changes in the descriptions of those found incapable may reflect a shift in diagnostic vocabulary or greater caution in acknowledging partial capacity, rather than any great change in the types of person who were found incapable. Furthermore, many post-war archives are not yet open for inspection, and those that are include an over-representation of older individuals living in a hospital or nursing home, with what were described as severe memory defects and confusional states.^4^ A fuller archive may therefore reveal more in time. That said, a number of factors suggest the arrival of a more cautious approach to incapacity.

Firstly, the CoP was challenged on its interpretation of incapacity in 1953, seemingly for the first time in nearly fifty years (*Re Gilchrist* [1907]). Of course, there may have been unrecorded challenges over the intervening years, but it was not a sufficiently important point to be mentioned in textbooks or formal enquiries into CoP work, and most of the cases in which the Visitors were called to give evidence concerned the testamentary capacity of a deceased individual, and not the capacity of the living (TNA LCO 2/5714). The 1953 case of EAKM therefore marks something of a turning point. M had been found incapable in 1941, and applied in the early 1950s to be restored to her property. The Master rejected her application and she appealed his decision; the case was unreported, but recorded in textbooks and archival notes because of its findings in relation to the disclosure of Visitors' reports (Hunt & Phillips, 1954, p. 83 (and later editions); TNA LCO 2/5714). On appeal, the Lords Justices found in her favour on the grounds that, although she still experienced certain delusions, she was sufficiently recovered to manage her own affairs. Her delusions concerned the past conduct of a member of her family and seem to have been taken by the Visitors and Master alike as sufficient evidence of continuing incapacity. Her successful appeal gave a strong indication that the actual impact of any infirmity or delusion upon the decisions to be made should henceforth be considered more closely.

This case coincided with renewed governmental attention to mental illness in the form of the Percy Commission, convened in 1954 to enquire into ‘the law relating to mental health and mental deficiency’. Its report formed the basis of the 1959 Mental Health Act (HMSO, 1957). The Commission did not see the CoP as within its terms of reference, but it did hear evidence from the Master and Visitors and made some passing comments. Notably, the Commission's report warned the CoP (and other medico-legal practitioners) not to assume too quickly that individuals who were detained as persons of unsound mind were necessarily incapable of managing their affairs (HMSO, 1957, pp. 290, 292–293). As I have written elsewhere, the CoP was perhaps more flexible in practice than this suggested (Weston, 2019), but nevertheless the 1959 Act made it absolutely clear that hospitalisation should not mean disenfranchisement. Section 101 stipulated that there was to be an ‘objective test of mental capacity in every case’ coming before the CoP. (Hunt & Reed, 1961, pp. v, 4). This encouragement to avoid CoP interventions unless strictly necessary may well have prompted a narrower view of what constituted incapacity, focusing more upon state of mind than a wider sense of perceived need or benefit.

This is not to say that the individual's wider situation was ignored in the second half of the century. Perhaps the most frequently cited judgment from the ‘old CoP’ is *re CAF,* which affirmed that incapacity was a matter of degree, related to ‘the circumstances, including… the complexity and importance of the property and affairs which he has to manage or administer’ (Hunt & Reed, 1964). In this particular case, it was the size of the individual's estate in combination with her difficulties with independent thought and communication that rendered her incapable in the eyes of the CoP, even though it was generally agreed that she was able to come to a ‘reasoned judgment’ of her own in relation to simple matters. *Re CAF* was immediately recognised as significant and described in a supplement to the ninth edition of *Heywood & Massey's Court of Protection Practice*, suggesting that the assessment of incapacity was by this time recognised more widely as potentially difficult and controversial.

The circumstances surrounding *re CAF* were suggestive of another change in determinations of incapacity. F had suffered a series of strokes, impairing her speech and brain function. She was therefore typical of one of the groups that came to dominate the work of the CoP in the second half of the twentieth century: those with an acquired brain injury, usually the result of a road traffic accident, industrial accident, medical negligence, or as in this case, stroke (Lush, 2018). They were eventually second in number only to those with disorders related to aging, often described loosely as ‘senility’ or ‘senile dementia’ (TNA LCO 68/22, Law Commission, 1995, p. 21). These conditions replaced the mania, delusions, psychosis, melancholia, and ‘dementia’ – the latter used during this earlier period to refer to incurable mental disorder not necessarily related to age – which had made up the bulk of the CoP's earlier caseload (Weston, 2019).

This shift reflected both changing prevalence and changing perception. More people were living longer, and research into and awareness of dementia began to attain a much higher profile. Such research remained generally pessimistic about anything other than a rapid cognitive decline amongst those so diagnosed (Hilton, 2015; Wilson, 2017), meaning that eventual incapacity was consistently seen as inevitable. Medical interventions were also enabling individuals to survive events involving brain injury that would, in earlier decades, have proved fatal, and some of these injuries prompted payments of compensation which would demand CoP involvement. At the same time, the introduction of an array of new pharmacological interventions for mental illness from the 1960s onwards, and the move to reduce institutional care, both coincided with the emergence of campaigns focusing upon the rights of those diagnosed with mental illness or what was then still called mental defect (National Council for Civil Liberties, 1951; United Nations, 1971). Those with all but the most severe difficulties were increasingly positioned as able to benefit from treatment, to live in the community, and in full possession of rights that should only be removed in the most extreme situations. The bar for incapacity in cases of mental illness and learning disability was, perhaps, being raised.

## 5 Managing property

Having determined that an individual was incapable of managing their affairs, it fell to the CoP to see that their property was looked after. The extent of this property in a typical case underwent a dramatic change in the early twentieth century: the CoP began to deal with many more small estates. This marked a change from the nineteenth century, when, as Akihito Suzuki has observed, lunacy inquisitions concerned almost exclusively the very wealthy (Suzuki, 2006). Evidence to the 1877 Select Committee on Lunacy Law hinted at the affluence of those found to be lunatics through the process of inquisition, with one expert witness reporting that ‘a good many’ received an annual income of £3000 or more (over £200,000 today) (HMSO, 1877, p. 57). The rich continued to be represented, with the CoP consistently engaged in managing large houses and country estates, five and six-figure inheritances, and sometimes much more income than could be spent (TNA J127/189; J92/25; J92/22; J92/77). Yet, much more modest estates came to dominate, numerically at least. More of the population may have owned some property and had a bank or savings accounts by the 1920s, but such accounts often held a very small balance (Atkinson, 2000), and even a small balance would require legal intervention if its owner appeared to lack capacity.

This use of the CoP was encouraged by the much easier and cheaper option of applying for a receiver instead of an inquisition in lunacy. A Royal Commission on the Care and Control of the Feeble-Minded noted the impact of this in 1908, reporting that the CoP had in recent years handled 606 cases involving income of less than £20 per year (equivalent to around £1500 today). This constituted 16% of all its cases (HMSO, 1908, p. 260). The proportion of small estates continued to rise, and in the 1920s the CoP responded to demand by introducing a streamlined application process for individuals whose annual income did not exceed £100 (around £4000 today). Soon afterwards, inspired by a recommendation within the 1926 Report of the Royal Commission on Lunacy and Mental Disorder for a ‘poor persons’ correspondence department’ (HMSO, 1926), a Personal Application Division was set up to help ‘applicants to obtain an Order for the appointment of a Receiver as cheaply and as expeditiously as possible’ without instructing a solicitor (TNA J92/103). Within just five years, half of all the applications were made through this Division (TNA LCO 4/50). Although some in the legal community felt that the Personal Application Division was actively (and improperly) advertising its services and encouraging applicants who could very well afford a solicitor to proceed unrepresented (TNA LCO 4/50), this was likely borne of broader frustrations with the CoP and anxiety amongst solicitors about loss of business. In the archives, the overwhelming majority of personal applications were indeed concerned with small estates. Many of these could be dealt with without appointing a receiver, if there was unlikely to be any ongoing management required (TNA J92/216; J92/219; J92/197), but even small funds often required some oversight and administration. By 1975, one memo recorded that 80% of the CoP's open cases involved annual incomes of under £1000 (about £8000 today) (TNA LCO 65/178).

The rise in the proportion of CoP cases that involved small estates coincided with another quiet change in CoP work: staff began to enquire more closely into the minutiae of their case files. At least some of this was prompted by anxiety about dishonesty and financial abuse. ‘I have been shocked by the number of cases of deliberate fraud’, Master Methold reported in 1934, after seven years in post (TNA LCO 4/50). To some extent, this more active engagement reflected demand from receivers, solicitors, and those found incapable. Complaints or requests were investigated more thoroughly from the 1920s onwards (cf TNA J92/20 and J92/28), with the Lord Chancellor's Visitors often called upon to contribute towards such investigations (TNA J92/300; J92/313; J92/34; J92/118). When asked directly whether the CoP was adopting a more interventionist approach, senior staff were quick to deny that they undertook too much detailed inspection of receivers' activities. They did, however, acknowledge more engagement with case files: in 1934, Master Methold described ‘a closer scrutiny’ of cases taking place, while Assistant Master Poyser implied that they would no longer aim to leave a person's affairs essentially unchanged wherever possible during a receivership (TNA LCO 4/50). Scrutiny and active management were becoming the order of the day, but were accompanied by backlogs of work and complaints of delay.

A shift in the CoP's activities is also suggested by the contents of the leading practitioner's textbook, *Heywood & Massey's Court of Protection Practice* (Heywood & Massey, 1900, and subsequent editions). This went through many editions from 1900 onwards, and included senior CoP staff amongst its authors from 1920. Early editions focused upon the procedure for the appointment of a committee or receiver, mentioning only briefly the decisions or activities involving the CoP that might occur subsequently. This balance was reversed in later editions, which saw new chapters on replacing receivers, conveyancing, litigation, appeals, marriage, settlements, and annual accounts. Perhaps at one time, the CoP's primary responsibility had been at the moment of appointing a receiver and it could then simply wait for receivers to apply for any further orders if necessary, but by the 1930s, this was only the beginning of its involvement in an individual's affairs. Especially where no solicitor was instructed, this could be time consuming and laborious for all involved.

The CoP today is criticised for failing to strike the right balance: for failing to identify and act on cases of fraud or exploitation, and also for imposing unduly heavy burdens upon receivers or interfering too closely. Its difficulties here are longstanding. Its efforts to look more closely at receivers' activities were not always welcomed or understood. Most receivers were family members, and increasingly, they dealt directly with the CoP with little or no legal or financial advice. Mr. Arthur Day's wife and receiver Louisa Day was sometimes impatient in her response to the CoP's biannual enquiry as to how she was spending her husband's small income. ‘I feel I have quite done a good part by him as the [hospital] Attendant would tell you if you care to inquire’, she wrote. ‘I never miss the visit & never shall while my Husband is a patient there he has been an invalid 12 years last October so I think I've done my part’ TNA (J92/58). Walter Marsh's sister and receiver, Mrs. Jane Thornley, was regularly confused and angered by the CoP's occasional requests for information, and wrote in simple terms: ‘I dont understand these forms you keep sending me to fill in’. She was frustrated to learn that only Mr. Marsh's weekly income of 10 shillings (£25 today) was available to her, not the capital, and annoyed by regular enquiries from the CoP as to how this weekly sum was spent (TNA J92/114). Margaret Fowler's father was also angered by the ‘high-handed way’ in which the CoP approached his family's affairs, having assumed that he as receiver would be given the freedom to deal with his daughter's Post Office savings as he saw fit (TNA J92/113; see also J92/207).

This irritation occasionally led to appeals against CoP decisions regarding income and expenditure. Any person affected by the decisions of the CoP could appeal to a Judge in Lunacy, although this right was infrequently exercised (TNA LCO 4/50; LCO 4/55). Master Methold's first two appeals were, in his words, ‘one case where the patient's wife was his Receiver and asked to be given the whole of his fortune’, and ‘a professional Receiver’ seeking ‘a larger remuneration than I had given to him’ (TNA LCO 4/50). Other appeals concerned payments to family members for automobile outings, the cost of which consumed nearly half the available income (TNA J92/57), and the payment of income to a local authority to cover the individual's maintenance (*re TRM* [1939]). Gifts and settlements proved particularly difficult. Late nineteenth century cases showed a reluctance to provide discretionary financial support to the wider family of someone found incapable, unless there were clear indicators of a prior intent to do so. Such questions had to be based on what the individual would have done if they were able to make the decision, but the Lunacy Office required evidence of the individual's specific intentions regarding each payment or settlement. It was not prepared to extrapolate from past patterns of behaviour to new situations, nor to guess at an individual's likely views even when the proposed gifts seemed reasonable. In one case Cotton LJ remarked that ‘it is not our business to deal benevolently or charitably with the property of the lunatic’, and added that applications for payments of an altruistic kind, such as a small but regular gift to an impoverished niece or nephew, ‘ought to be discouraged rather than encouraged’ (*Re Darling* [1888]; see also *Re Frost* [1870]). This was part and parcel of a desire to preserve the incapable individual's estate unchanged so far as possible, which began to fade as a more interventionist approach to estate management took over.

In the early twentieth century, there were signs of a different approach to estimating what someone found incapable would have done, with a more imaginative reading of their wishes. This was enabled by s.171 of the Law of Property Act 1925 and encouraged by changes to intestacy provisions brought about by the Administration of Estates Act of the same year, although such decisions harked back to an earlier judgment from 1816 (*Ex parte Whitbread* [1816]; Skinner, 1999). The basis on which to decide what someone would have done was argued in 1927, in a case concerning a settlement in favour of a Miss Freeman's second cousins. The Crown's case was in keeping with the older view: ‘It is a matter of pure speculation whether the lunatic would have left her property to second cousins’, and so there should not be any settlement in their favour. But this did not find support. The Court was able to imagine that Miss Freeman, if she were aware of all the circumstances that had arisen since her incapacity, would be likely to choose her second cousins as the beneficiaries of her property after her death (*re Freeman* [1927]). Also relying on the Law of Property Act and its requirement to avoid ‘injustice’, another settlement in 1941 directed funds to various Masonic charities. This was because the person found incapable had come into an inheritance, the terms of ‘which indicated that the testator expected that the patient would devote such funds to such objects’ (*re TCW* [1941]). The court was willing to imagine, without any specific evidence to this effect, that the recipient of this windfall would have wanted to honour such a wish.

This is similar to what is now described as a substituted judgment approach, where there is an attempt on behalf of someone found incapable to make the choice that most closely represents the decision they would themselves have made. When mental capacity law came under scrutiny in the 1990s, the substituted judgment approach was contrasted with a ‘best interests’ approach, in which the decision to be taken was ‘that which the decision-maker thinks is best for the person concerned’ (Law Commission, 1991, para 4.22). The Law Commission preferred this ‘best interests’ approach, albeit modified with elements of substituted judgment to reduce its paternalistic overtones (Law Commission, 1995). Broadly speaking, this was adopted in the MCA, 2005. As has been noted elsewhere, the original form of substituted judgment envisaged in the Whitbread case of 1816 paid little attention to the individual whose decision was being imagined and their personal views or foibles: instead, the court considered in the abstract what a reasonable person would be likely to decide (Szerletics, 2012). These CoP cases from the mid-twentieth century are similar, in that relatively little attention is paid to the incapable person as an individual. In Miss Freeman's case, the only personalised comment about her was to note that she had made a number of wills before her incapacity. This was taken to suggest that she would have wanted to make some testamentary disposition in the circumstances that had arisen, instead of letting her estate pass to the Crown. Later cases also showed little inclination to reflect on the individual whose affairs were in question, but rather, to consider the decisions that might be made by a hypothetical reasonable person in the same position (*re RHC* [1962], *Re L (WJG)* [1965]). In practice, then, this mode of substituted judgment delivered relatively de-personalised decisions, against which the ‘best interests’ approach proposed in, 1995 could be contrasted. We might therefore see the move towards ‘best interests’ as a reaction to the implementation of the substituted judgment approach in these older CoP cases.

Gifts and settlements continually featured prominently as a difficult issue for the CoP for two main reasons. Firstly, rising death duties over the mid-twentieth century incentivised the wealthy to disperse at least some portion of their fortune amongst relatives during their lifetime, and receivers sought in many cases to replicate this with the property of those found incapable. Secondly, the 1959 Mental Health Act placed the Court's jurisdiction on a new statutory footing, which led to a small flurry of reported cases to provide interpretations of key terms from the Act including ‘family’ and ‘benefit’, contemplating the basis upon which the patient's likely wishes should be imagined. In broad terms, discretionary settlements for the purposes of estate planning were permitted, including the avoidance of death duties, the production of equality between family members, and overcoming intestacy provisions to secure the interests of illegitimate children (*Re CWM* [1951]; *Re CEM* [1956]; *Re AHS* [1956]; Re CCHR [1957]; *Re RHC* [1962]; *Re CEFD* [1963]; *Re L (WJG)* [1965]; *Re DML* [1965]; *Re TB* [1967]). It was, therefore, somewhat disingenuous for the preface of the ninth edition of *Heywood & Massey* to record that the introduction of statutory will making in 1969 had disrupted more than six centuries of the CoP's duty ‘not to interfere with rights of succession’ (Hunt, Reed, & Whiteman Reed & Whiteman, 1971, p. v). Some interference had become acceptable, as the CoP engaged more closely with receivers and the lives of its charges. This no doubt paved the way for statutory wills.

It was not only finances that interested the CoP. As the body with ultimate responsibility for all expenditure, it might be required to give approval for costly medical treatments and had some reason to enquire into living circumstances. This was in spite of the fact that it had no jurisdiction over matters of personal welfare except in those vanishingly rare circumstances before 1959 when a committee of the person had been appointed under the old inquisition process. Unsurprisingly, its interest in personal matters could lead to disagreements. Mrs. Emma Ward lived with her son, who had acted as her receiver following her nervous breakdown in 1924. Her accommodation was found ‘unsatisfactory’ by a Lord Chancellor's Visitor, and her son responded furiously to an ‘impertinent’ letter to that effect from the CoP. ‘My mother is a strong active woman who unfortunately at times will quarrel with any other woman who lives near her’, he explained.

When eventually they have had enough of her, my mother writes to your Department complaining of where she is living - and landladies & neighbours complain to me. This has been going on for over six years and has caused me to have to move three times…. Just get your Mr. Thompson to state in writing explicitly in what way the accommodation is unsatisfactory. His swaggering around in a large car has made my mother discontented.

With less evident anger, others found themselves addressing the CoP with details of daily living such as why they did not want a wireless, requests for new underwear, and permission to give wedding presents to family (TNA J92/39, J92/202; J92/41). In the 1970s, as in the 1930s, generalised concerns and complaints about the CoP suggested that it was going beyond its brief, ‘prying into matters which don't concern the jurisdiction of the court’ and creating excessive volumes of work for itself by trying to control receivers too closely (TNA LCO 65/178). This coincided with broader efforts in the 1970s to reduce legal controls over those with mental illnesses, ultimately leading to the accidental creation in the 1980s of a legal lacuna concerning personal decision-making on behalf of those lacking capacity (*Re F* [1990]).

Irritation flowed both ways. Flashes of annoyance in the archives suggest that CoP officials were sometimes less than impressed by receivers and their legal representatives. ‘This Affid[avit] is quite useless’, recorded Master Theobald in 1921, as part of a thoroughgoing critique of some professional correspondence coming into his office (TNA J92/34). ‘I have on my desk at this moment a glaring example of the apparent incompetence of a certain firm of solicitors’, retaliated Chief Clerk Gerard Mills, when faced with a complaint about the CoP (TNA LCO 4/47). Red flags about competence and reliability on the part of receivers encouraged particularly close attention. A bad-tempered spat ensued when Mrs. Bradley's daughter and receiver Constance asked to be given more of her mother's income for her own benefit. ‘The statement filed by the sol[icitor]s is an impudent affront to the authority of this M[aster]’, noted one official, after recording that Constance already took a larger portion of Mrs. Bradley's income than was spent on Mrs. Bradley herself. A clerk quickly contacted the hospital where Mrs. Bradley lived, to verify that Constance was telling the truth about visiting her mother regularly, and a full review of the various decisions so far made about Mrs. Bradley's estate was conducted. Constance was eventually reminded in ominous tones that she only received anything at all at the Master's absolute discretion. This discretion was not tested again (TNA J92/48). In other cases lay receivers overspent, muddled receivership money with their own, failed to deliver accounts on time or at all, behaved suspiciously, and sometimes disappeared altogether, leaving CoP staff to battle with sometimes chaotic situations as best they could (TNA J92/57; J92/18; J92/21; J92/315; J92/202). Receivers were often replaced, usually as a result of their own infirmity and age but sometimes for more worrying reasons: ‘where the receiver has not proved to be a fit and proper person… rendered unsatisfactory accounts or failed to account, lent the patient's money on mortgage’, and so on (Hunt & Phillips, 1954, p. 80). Recent commentary on the new CoP suggests that the timely identification of financial abuse remains a significant problem; the balance between effective control and undue interference remains difficult to strike (Terrell et al., 2018).

## 6 Survival and reform

The role and existence of the CoP was quietly questioned in the 1970s, for the first time since the start of the century. Sixty years earlier, the proposal in the air had been to hand over its duties to the Board of Control, which was responsible for mental hospitals and their patients. Henry Studdy Theobald, Master from 1907 to 1921, would happily have seen his office dissolved and this idea initially gathered some popularity, only to fall by the wayside amidst other aspects of law reform then to be firmly rejected by the end of his tenure (TNA LCO 4/47). To be sure, the CoP was still criticised as inefficient and expensive, but until the 1970s the proposed solutions – those implemented and those abandoned alike – addressed its processes, staffing, and funding, rather than the existence of the CoP itself. In short, there was no internal appetite or external pressure for radical change.

Unspecified internal restructuring and a larger budget were the solutions to its administrative problems in 1934 (TNA LCO 4/50), but this was a temporary fix. Over the ten years to 1948, staffing levels increased further by only 3% while the number of cases rose by 47% (TNA LCO 4/55; LCO 4/409). Workloads were overwhelming and solicitors persistently complained about delays, setting a pattern that would be followed for decades to come. One wrote in heated terms in 1947 about the CoP's ‘antediluvian procedure which originally seems to have been designed in Bedlam itself’ (TNA LCO 4/54). A decade later, the position was slightly better but still not ideal, and the report of the 1954 Enquiry into mental health law suggested that delays might be avoided if the property of those found incapable were managed by local authorities in the case of smaller estates (HMSO, 1957). This was energetically investigated but eventually shelved due to the ‘complicated legislation’ that would be required for a relatively small number of estates (TNA LCO 2/5701). Another option was to adopt the more hands-off approach to patients' estates that was rumoured to exist in Scotland, and a fact-finding mission to Edinburgh took place in 1967 but ended in disappointment: ‘the procedure is extremely expensive and I am very far from convinced that the patient gets such good service from a curator who is left to his own devices’ (TNA LCO 4/409).

Adding to its woes was the matter of cost. It was acknowledged that the CoP delivered a wide-ranging and extensive service, greater in scope than was found in other jurisdictions, but it was no longer as comfortably self-financing as it had once been. The problem was ‘those who want a Rolls Royce for the price of a Ford Cortina’ (TNA LCO 4/409): the CoP was the Rolls Royce of incapacity law, but its clients were no longer the extremely wealthy who could afford to pay for this. In 1908, it still showed a substantial annual surplus of £11,000, nearly 40% of its income from fees and charges on the estates it oversaw, but this did not last. Smaller estates meant smaller income, as fees were generally calculated as a percentage of the property being managed. In the case of particularly small estates, fees would be waived to avoid hardship. The first sustained losses in the books of the CoP were temporarily handled by an increase in the fees and charges in the early 1920s, which returned the CoP to profitability for the last time. A deficit became a permanent fixture from the 1930s until the 1990s, dented only slightly by further fee increases (TNA LCO 2/7695; LCO 65/178; LCO 68/22; House of Commons, 1991).

The proximity of the CoP to a welfare service meant that some subsidy from the Exchequer was largely (if not entirely) seen as acceptable, at least over the middle decades of the century (TNA LCO 2/7695).^5^ This proximity may also help to explain its survival. From the Liberal reforms of the early twentieth century through to the arrival of the NHS in 1948 and beyond, centralised services were being introduced for the benefit of the young, the old, the unemployed, and the unwell. A service to protect the property of those unable to protect it themselves could be seen as part and parcel of these schemes. But its financial demands also left it open to more criticism, especially as the welfare state itself as well as the costs of the Lord Chancellor's Office were squeezed in the 1970s. This was, it seems, an unhappy period for the CoP. Concerns persisted about the ‘the whole position of the Court of Protection, which seems to be generally inefficient, cumbersome and expensive’. The position of the Lord Chancellor's Visitors was criticised, as their qualifications and salary did not seem to tally with the nature of their work (TNA LCO 65/178). The Visitors themselves hinted at their dissatisfaction with the CoP (TNA LCO 65/17). Furthermore, and for the first time in many decades, there were sustained complaints and concerns aired by MPs and in Parliament, including calls for a formal enquiry (House of Commons, 1973). Complaints about CoP investment practices had first been aired in the 1950s (TNA LCO 2/5710), but this issue as well as the fees charged by the CoP was taken up with new energy.

In this context, the effective dissolution of the CoP and the office of the Lord Chancellor's Visitors was tentatively discussed. The idea was to place all judicial and audit work in the hands of the county courts, and the welfare work with social services. But this stalled when the CoP was excluded from the Review of the Mental Health Act in 1975. This followed a pattern set by the 1926 and 1954–57 enquiries into mental health law, which cast the CoP as something entirely separate. Efforts to reform mental health law focused very much throughout the twentieth century on questions of treatment and involuntary detention, and not the property, or property rights, of the mentally ill. This separation not only meant that the CoP largely evaded criticism and public attention, but also rendered its work somewhat unknowable, as comprehensive reviews and critiques were absent. The ‘work of the Court remains a mystery’, remarked one civil servant rather sadly in 1979 (TNA LCO 65/178).

This air of unknowability was aided by the veil of discretion which had been drawn over the vast majority of CoP activities throughout most of the twentieth century, with proceedings heard in camera. Perhaps the memory of those very public nineteenth century inquisitions lingered, or at least, the reluctance of the general public to air their personal, financial, and medical matters in open court was hypothesised. CoP cases were an exception to the principle that justice should be administered in public, because of ‘the quasi-paternal jurisdiction’ of the judge. When acting ‘in lunacy’, the House of Lords had found in 1913, ‘the Court is really sitting primarily to guard the interests of the ward or lunatic’, and not to decide a dispute, and so their primary duty was the lunatic's care and not to the public delivery of justice (*Scott v Scott* [1913]). It seemed to go without saying that care demanded silence. This practice was criticised by Ungoed-Thomas LJ in 1971 (*Re W (EEM)* [1971]), to little apparent effect, and it was only when the CoP became a court of record in 2007 that its judgements were published with any regularity. A tension between protecting individual privacy and avoiding accusations of sinister secrecy remains clearly in evidence today (Doughty & Magrath, 2016). The low profile of the CoP's decisions throughout the twentieth century may have facilitated its survival, allowing it to avoid too great a scrutiny from the general public or from more official inquiries into mental health law.

It may also have mattered to the outcome of discussion in the 1970s that the incumbent Master of the Court of Protection seemed less open to reform than his predecessor, with many memos reflecting anxiously upon his likely opposition. In this he was joined by the Permanent Secretary in the Lord Chancellor's Department, Sir Denis Dobson, who was notoriously averse to change (Anon, 1995). In the end, reform of the CoP fell to be considered as part of a wider scheme of reform across the Lord Chancellor's Department. The problem of the four very senior and costly Visitors was solved by replacing them with a panel of medical, legal, and general visitors, with the bulk of their work to be carried out by general visitors who did not require any particular qualifications (E. R. Taylor, 1982). The CoP then gained a Management Division by taking over the Official Solicitor's Receivership Division in 1983, but effectively lost this to the Public Trustee in 1986 and then saw all of its administrative responsibilities transferred to the new Public Trust Office in 1994. Yet its survival in essence, along with the extent of its existing and anticipated caseload and its resultant claims to expertise left it well positioned to provide a conceptual foundation when the Law Commission and the government surveyed the field of mental incapacity law in the 1990s (HMSO, 1997; HMSO, 1998; Law Commission, 1991, Law Commission, 1995). The ‘old’ Court of Protection was formally abolished but reborn as a superior court of record under the Mental Capacity Act, 2005, dealing not only with its pre-existing business of finance, powers of attorney, and statutory wills, but also personal and healthcare decisions for those who lack capacity.

## 7 Conclusion

It was not envisaged, when the jurisdiction of the old Court of Protection was adjusted and consolidated in 1890, that the seemingly modest changes therein would have such impact. Nor was it anticipated that so many people would eventually apply to it for help. A modified jurisdiction combined with changing social, economic, and medical contexts drove up the numbers of those found incapable upwards over most of the twentieth century, save for when the post-war welfare state alleviated some of the pressures that encouraged CoP interventions in the 1950s. These trends continue today, and have been further heightened by the changes introduced in the 2005 Mental Capacity Act; the future impact of ongoing shifts in social norms and attitudes, welfare and care services, medicine, and the wider economy remains to be seen.

A growing caseload and wider variety of patients and receivers in the first half of the century was met with more active involvement on the part of the CoP in the management of the affairs of those found incapable. This generated its own problems in the form of costs, delays, and sometimes testy relationships with receivers and professional advisers, but led to new approaches and case law, particularly regarding gifts, settlements, and substituted judgements. It also prompted the identification of more cases of fraud or mismanagement. Striking the right balance between identifying dishonesty or abuse, and placing overly heavy demands on those acting on behalf of others, remains a difficulty today. It is also still the case that the vast majority of the workload concerning those found incapable is uncontroversial, uncontested, and settled without the need for a formal hearing. In the contemporary Court of Protection, decisions regarding health and welfare or deprivation of liberty are much more likely to be contentious than decisions regarding property and affairs (Ruck Keene et al., 2019), and the obsolescence of the inquisition process in the early twentieth century meant that health and welfare was not for the most part within the ‘old’ CoP's jurisdiction. The issues coming before the Court today are therefore different in some respects, but the bulk of its work – appointing deputies and issuing one-off orders in relation to property and affairs, appointing replacement trustees, executing statutory wills, and so on, all with a careful eye on the individual circumstances of each case – is a clear continuation of its former role.

This is not to say that the activities of the CoP have not changed at all. Attention to its history reveals that it was not quite the same kinds of people that have been found incapable, over the years. As the category of ‘lunacy’ fell by the wayside in the early twentieth century and ideas of mental illness shifted to include a wider variety of mental states, the idea of incapacity also expanded to include the elderly, the frail, and those who were vulnerable in the face of their own impulsiveness or pressures from others. For a period of time, at least, personal circumstances and relationships were just as important as medical diagnoses. The middle of the twentieth century perhaps saw the greatest variation amongst those found incapable, with these ‘borderline’ cases of vulnerability or advanced age co-existing in the CoP's casefiles alongside severe and moderate psychiatric illnesses and a growing variety of other conditions. There are signs that this shifted again in the second half of the century, with a narrower conception of incapacity emerging and a renewed attention to medical diagnoses and classifications. By the 1980s, cases of dementia and acquired brain injury had come to dominate the CoP workload, and this remains the position today. Over the course of the twentieth century, then, the typical case coming before the CoP changed from one concerning a very wealthy, middle-aged individual detained as a person of unsound mind and diagnosed with mania or delusions, to an older person of unremarkable means, suffering from dementia and cared for at home or in a nursing facility. In addition, the redesign of the Court of Protection in the 2005 MCA has meant that it now deals with more individuals with learning disabilities than before, under its new jurisdiction over health and welfare matters. Yet, fluctuations over the course of the century in who was being found incapable demonstrate that change is not always linear, and that interpretations of incapacity can expand as well as contract.

In spite of its considerable power, the substantial number of people affected, and its evident struggles over the years with efficiency and the proper extent of its responsibilities, the CoP proved remarkably resilient. In large part this was down to the absence of any external pressures for change, with issues of psychiatric treatment and involuntary detention occupying the minds of those concerned with mental health law and its reform. It is telling that, even when mental incapacity finally became a live issue in the 1990s, this was not propelled by concerns regarding the CoP but rather, by the lacuna in the law concerning capacity to consent to medical treatment. We might see this as evidence of success on the part of the CoP: there were no scandals or crises to prompt action, and it was sufficiently flexible and responsive to accommodate changing attitudes towards mental illness and money alike. We might also pause to wonder why financial protections or abuses attracted so little attention, for the most part, and to reflect on this legacy for the management of incapacity today.

## Financial support

This work was supported by the Wellcome Trust [grant number 209884/Z/17/Z].

## Declaration of Competing Interest

None.

## Appendix A Cases

In the matter of A (A Patient, now deceased), [2018] EWCOP 16

Masterman-Lister v Brutton & Co, [2002] EWHC 417

Re AHS [1956] (unreported)

Re Browne [1894] 3 Ch 412

Re CCHR 1957 (unreported)

Re CEFD [1963] 1 W.L.R. 329

Re CEM [1956] (unreported)

Re CWM [1951] Ch 714

Re Darling [1888] 39 Ch 208

Re DML [1965] 1 Ch 1133

Re F (Mental Patient: Sterilization) [1990] 2 AC 1

Re Freeman (1927) 1971 H&M

Re Frost [1870] 5 LR-Ch 699

Re Gilchrist [1907] 1 Ch 1

Re Hinde, ex parte Whitbread [1816]

Re L (WJG) [1965] Ch 135

Re RHC [1962] 1 WLR 1095

Re TB [1967] Ch 247

Re TCW [1941] (unreported)

Re TRM [1939] 1 Ch 260

Re W (EEM) [1971] Ch. 123

Re Walker [1905] 1 Ch 160, 174

Scott (otherwise Morgan) v Scott [1913] AC 417

## Appendix B List of Parliamentary debates mentioned

Hansard, HC (Written answers) 5 December 1974 vol 882 col 601-5

Hansard, HC (Written answers, 2 May 1991, vol 190

Hansard, HC Debate, 1 March 1973, vol 851 col 1833

## Footnotes

1 Relevant large-scale research projects include the *Essex Autonomy Project* <https://autonomy.essex.ac.uk/>, *Judging Values and Participation in Mental Capacity Law* <http://www.icpr.org.uk/judging-values-and-participation-mental-capacity-law>, *Mental Health and Justice* <https://mhj.org.uk/>, and *Welfare Cases in the Court of Protection* <https://sites.cardiff.ac.uk/wccop/>. Special issues of journals include ‘The Mental Capacity Act, 2005 – Ten Years On’, *Medical Law Review*, 24.3 (2016) and ‘Mental Capacities and Legal Responsibilities’, *International Journal of Law and Psychiatry*, 40 (2015).

2 Case files can be found under catalogue references J127 (records of the Official Solicitor) and J92 (records of the Court of Protection), at The National Archives, London (hereafter TNA). Administrative records are scattered within the records of the Lord Chancellor's Office, catalogue reference LCO.

3 This and subsequent currency conversions are very approximate, and use the online tool available at <http://www.nationalarchives.gov.uk/currency-converter>.

4 This imbalance is clear when considering, for example, that 65% of all those under the care of the Court of Protection lived at home in the early 1970s (TNA LCO 65/178), but the open archive contains only two such examples: TNA J127/188 and J92/300.

5 For a note of dissent, see the letter from Cartwright Sharp of the Law Commission to Sir Hume Boggis-Rolfe of September 1967, in TNA LCO 4/409.
